# Lipidomics Analysis Reveals Efficient Storage of Hepatic Triacylglycerides Enriched in Unsaturated Fatty Acids after One Bout of Exercise in Mice

**DOI:** 10.1371/journal.pone.0013318

**Published:** 2010-10-13

**Authors:** Chunxiu Hu, Miriam Hoene, Xinjie Zhao, Hans U. Häring, Erwin Schleicher, Rainer Lehmann, Xianlin Han, Guowang Xu, Cora Weigert

**Affiliations:** 1 Division of Endocrinology, Diabetology, Angiology, Nephrology, Pathobiochemistry and Clinical Chemistry, Department of Internal Medicine, University Hospital of Tuebingen, Tuebingen, Germany; 2 CAS Key Laboratory of Separation Science for Analytical Chemistry, Dalian Institute of Chemical Physics, Chinese Academy of Sciences, Dalian, China; 3 Division of Bioorganic Chemistry and Molecular Pharmacology, Department of Medicine, Washington University School of Medicine, St. Louis, Missouri, United States of America; 4 Paul Langerhans Institute Tuebingen, Member of the German Center for Diabetes Research (DZD), Tuebingen, Germany; University College Dublin, Ireland

## Abstract

**Background:**

Endurance exercise induces lipolysis, increases circulating concentrations of free fatty acids (FFA) and the uptake and oxidation of fatty acids in the working muscle. Less is known about the regulation of lipid metabolism in the liver during and post-exercise.

**Methodology/Principal Findings:**

We performed an ultra fast liquid chromatography-mass spectrometry (UFLC-MS) based lipidomics analysis of liver tissue samples obtained from C57Bl/6J mice immediately after a 60 min treadmill run of moderate intensity, and after 3 h of recovery. The PLS-DA scores plot for 115 quantified lipid molecular species revealed a clear separation of the hepatic lipid profile of sedentary from recovering mice, but not from mice immediately after running. 21 lipid species were considered to be most responsible for the difference in the hepatic lipid profiles, including 17 triacylglycerides (TG), one lysophosphatidylcholine (LPC) and three phosphatidylcholines (PC). TG species were found to be more abundant in the recovery phase, while PC species were decreased. The degree of accumulation of individual TG species correlated well with the amount of theoretical energy stored whereas no increase was found for TG species containing only saturated or one monounsaturated fatty acid. Total liver TG content as assayed by an enzymatic method was increased to 163% in the recovery phase, while it was significantly decreased in skeletal muscle by the exercise bout and remained less in the recovery phase. Results from fasted and refed mice indicate that fasting-induced lipolysis was associated with a pronounced accumulation of hepatic TG, which is reversed by refeeding for 5 h. Thus food intake per se did not elevate hepatic TG.

**Conclusion:**

These data indicate that high availability of FFA induced by endurance exercise or fasting resulted in a transient hepatic TG accumulation, while muscle TG content was decreased during exercise presumably due to increased muscle fatty acid oxidation.

## Introduction

Fatty acids are the major fuel during prolonged moderate intensity exercise. The availability of plasma free fatty acids (FFA) is markedly increased by the action of hormone-sensitive lipase and adipose tissue triacylglyceride (TG) lipase on the fat depots in adipose tissue and skeletal muscle [1], [2]. During exercise the relative proportion of FFA uptake in the working muscle is substantially increased [3], [4] and FFA are oxidized to provide energy or reesterified and incorporated into intramyocellular TG [5]. The liver has a central function in lipid metabolism by repartitioning FFA derived from body fat stores and integrating dietary FFA into whole body fuel oxidation and energy storage via secretion of lipoproteins. However its role in the process of FFA supply and tissue redistribution during and after exercise is not well characterized.

Studies both in rodents and humans provide evidence that the hepatic tissue concentration of TG is influenced by regular exercise. Training of rodents shows that enhanced physical activity prevents high-fat diet-induced hepatic steatosis when performed concurrently with the diet [6], [7] or thereafter [7]. Voluntary wheel running reduces the hepatic TG content in Otsuka Long-Evans Tokushima Fatty rats, a rodent model for obesity and type 2 diabetes [8]. These lipid-lowering effects of training could be absent in young, non-obese rodents [9]. Application of the noninvasive proton magnetic resonance spectroscopy (^1^H-MRS) to quantify liver fat in humans reveals that regular exercise training leads to the reduction of hepatic lipid content even in the absence of weight loss [10]–[12]. Of note, liver fat shows the highest percentage of reduction in response to exercise interventions compared with visceral and subcutaneous lipid stores [13], [14].

Further training-related effects on hepatic lipid metabolism are the regulation of lipoprotein secretion from the liver leading to a reduction of the postprandial concentrations of plasma TG found both after one single bout of acute exercise [15]–[18] and after regular exercise training [6], [19]–[22]. The contribution of distinct mechanisms leading to this hypotriglyceridemic effect of exercise are not completely understood, but it is predominantly a decrease in the very low density lipoprotein (VLDL)-TG fraction [18], [23], [24]. This decrease has been attributed to an increased plasma clearance rate of VLDL-TG [25]–[28] and a reduced VLDL-TG secretion, which has been described in humans [29], and studies in rats have provided further evidence for that [30], [31].

Thus, beneficial effects of regular physical activity on total liver fat and plasma lipids in humans or rodent obesity models are well documented, but the response of hepatic lipids to acute exercise are less clear. We hypothesized that the investigation of the hepatic lipid profile after one single exercise bout could not only give insights in the exercise-induced adaptive mechanisms of hepatic TG storage but also reveal further impact of exercise on other lipid species found in the liver.

We applied here a recently established reversed-phase ultra fast liquid chromatography−mass spectrometric (UFLC−MS) method [32] for the lipidomics analyses of hepatic lipids of mice after one single bout of treadmill exercise. We combined the hepatic lipid profiling with conventional determination of plasma and tissue TG content. The results indicate decreased choline phospholipid and increased TG levels in the liver after acute exercise, with a more efficient storage of TG molecular species containing unsaturated acyl chains.

## Results

### Plasma parameters

**Figure 1:**
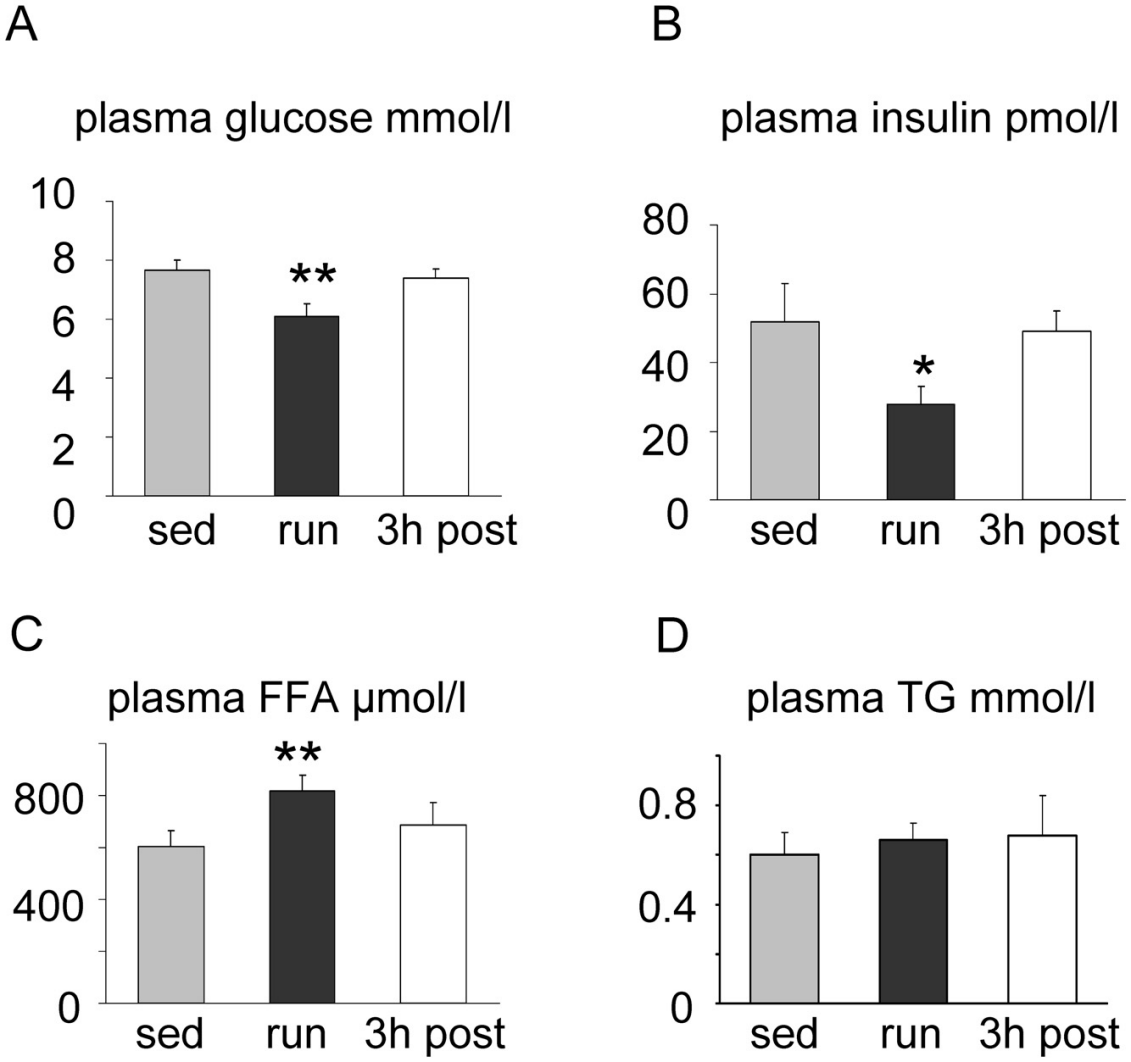
Plasma parameters. Plasma concentrations are shown for glucose (A), insulin (B), FFA (C), and TG (D) of sedentary (sed), immediately after exercise (run), and recovering (3 h post) mice (n = 12, mean ± SEM; * p<0.05; ** p<0.01 vs. sed).

The running protocol applied to the mice was designed to provide a strong, but non-exhaustive metabolic stimulus, resembling intensive endurance exercise. Immediately after the exercise bout, plasma FFA levels were significantly increased, and plasma glucose levels as well as plasma insulin concentrations were significantly decreased (Fig. 1A–C). After 3 h of recovery with free access to food in the first 2 h, these metabolic parameters were similar to those in sedentary mice. Plasma TG levels were not different in either condition (Fig. 1D).

### Hepatic lipidomics reveals differences between recovering mice and sedentary mice

A total of 115 lipid molecular species were identified and quantified by the current UFLC−MS-based lipidomics platform with phosphatidylcholines (PC) and TG as most abundant classes (summarized in Table 1; the complete data are available as Supporting Information in Table S1).

**Table 1:** Summary of detected lipid classes.

Lipid class	Number of detected lipid species	Sum of all detected species		
		sedentary	0 h (run)	3 h post
		(mean ± SD, µmol/g)	(mean ± SD, µmol/g)	(mean ± SD, µmol/g)
LPC	6	0.462±0.063	0.408±0.113	0.365±0.091
PC	24	4.542±1.024	5.344±1.115	4.501±1.281
PE	14	0.634±0.154	0.739±0.261	0.626±0.184
SM	7	0.352±0.039	0.367±0.116	0.307±0.048
DG	5	0.129±0.029	0.138±0.021	0.126±0.037
TG	59	5.656±1.497	6.063±1.187	6.900±1.850

**Figure 2:**
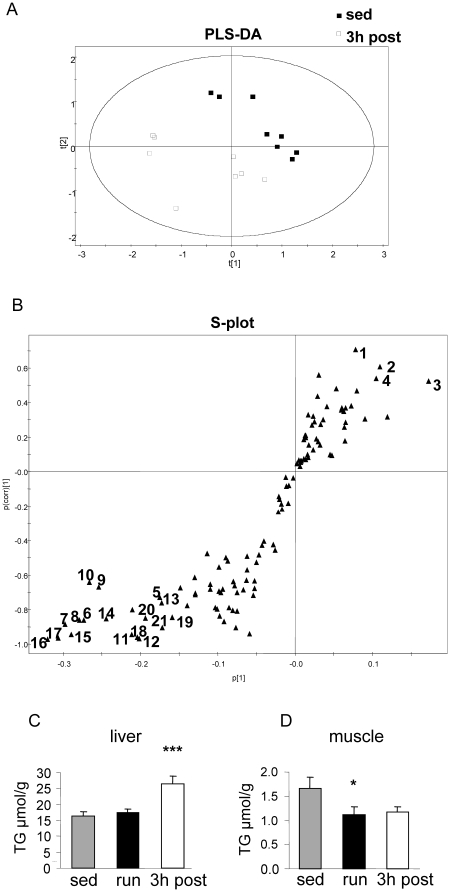
Hepatic lipidomics reveals differences between recovering mice and sedentary mice. Partial least squares discriminant analysis (PLS-DA) of hepatic lipidomics data was applied to differentiate the recovering (3h post) mice (n = 8) and the sedentary mice (n = 8) with 115 identified lipid species included in analysis as variables. (A) Scores plot of T [2] vs. T [1]. Each data point on the plot represents the individual hepatic lipid profile of one animal analyzed by UFLC-MS; (B) S-plot of the loading of PLS-DA component 1 (*p* 1; modeled variance) plotted against the modeled correlation [*p* (corr)], used to identify lipid metabolites most discriminatory for the specified classification. Each data point on the plot represents one out of 115 lipid molecular species. Lipids marked by numbers (see Table 2 for the corresponding lipids) were defined as potential discriminators with variable importance in the projection values greater than 1 in at least one of the two groups. (C,D) TG concentrations as determined by an enzymatic analysis as described in methodsper g of tissue are shown for liver (C) and quadriceps muscle (D) of sedentary, immediately after exercise (run) and recovering (3 h post) mice (n = 12 (liver), n = 4 (muscle), mean ± SEM; * p<0.05; *** p<0.001 vs. sed).

Based on the hepatic lipid metabolite pattern the PLS-DA scores plot revealed no separation of sedentary mice and mice immediately after the run (i.e. the number of components in the autofit model was zero), but a clear separation between recovering mice and sedentary mice was achieved (Fig. 2A). To ensure that the calculated model of the recovering mice vs. sedentary mice is reliable and the observed clustering has not been obtained by chance, we performed an internal validation using 7-fold cross-validation [33]. The calculated goodness of fit (R2Y) was 0.991 and the goodness of prediction (Q2Y) was 0.886 which underlines the robustness of the model. In addition, a response permutation test was carried out. The R^2^Y-intercept and Q^2^ -intercept were 0.385 and -0.151, respectively, showing that the model is not over-fitted [33]. The S-plot, visualizing both the covariance and correlation between the variables and the modeled class designation, highlighted the lipid species which contributed most to separation of recovering mice from sedentary mice (Fig. 2B). In combination with PLS-DA scores plot and the variable importance in the projection (VIP) values of all lipid ions included in the dataset for multivariate statistical analysis, 21 hepatic lipid metabolites (numbered in Fig. 2B) were considered to be most responsible for the differences between recovering mice and sedentary mice. Among these 21 affected lipids, 4 choline phospholipids were decreased whereas 17 TGs were increased in the liver of recovering mice (Table 2). TG (50∶3), TG (52∶6) and TG (54∶5) showed the highest relative increase.

**Table 2:** Lipid species responsible for the discrimination of the hepatic lipid profile of recovering mice from sedentary mice screened by S-plot from PLS-DA.

No.	m/z	t_R_ (min)	Lipid species	Ion adduct	3 h post vs. sed
					Up/Down (%)
1	544.342	2.1	LPC (20:4)	H^+^	−27
2	788.619	11.4	PC (36:1)	H^+^	−23
3	784.587	9.9	PC (36:3)	H^+^	−11
4	812.618	10.9	PC (38:3)	H^+^	−23
5	818.726	17.0	TG (48:3)	NH_4_^+^	30
6	850.789	19.2	TG (50:1)	NH_4_^+^	13
7	848.773	18.5	TG (50:2)	NH_4_^+^	15
8	846.759	17.8	TG (50:3)	NH_4_^+^	86
9	876.805	19.3	TG (52:2)	NH_4_^+^	11
10	874.789	18.7	TG (52:3)	NH_4_^+^	23
11	872.775	18.2	TG (52:4)	NH_4_^+^	25
12	870.760	17.5	TG (52:5)	NH_4_^+^	30
13	868.744	16.8	TG (52:6)	NH_4_^+^	50
14	904.837	20.2	TG (54:2)	NH_4_^+^	12
15	902.820	19.5	TG (54:3)	NH_4_^+^	24
16	900.805	18.8	TG (54:4)	NH_4_^+^	27
17	898.789	18.1	TG (54:5)	NH_4_^+^	43
18	896.774	17.6	TG (54:6)	NH_4_^+^	40
19	932.868	21.0	TG (56:2)	NH_4_^+^	21
20	930.851	20.3	TG (56:3)	NH_4_^+^	30
21	928.836	19.6	TG (56:4)	NH_4_^+^	31

In order to further investigate quantitative changes of lipids in the recovery phase as compared to the sedentary controls, evaluation of statistical significance for all identified 115 lipid species were carried out across the groups (Table 3). It was observed that the lipid species TG (50∶3), TG (54∶5), TG (54∶6), TG (54∶7), TG (56∶4), TG (58∶6) and TG (58∶10) were significantly increased whereas lipids of PC (36∶1), PC (38∶3), PC (40∶4) and DG (34∶1) were significantly decreased in livers of mice in the recovery phase versus sedentary controls. Only PC (36∶1) and PC (36∶3) detected by VIP analysis were among the eight most abundant PC species which together comprise approximately 70% of all detected PCs. The majority of the abundant PCs was unchanged in the recovery phase (Table 4). The eight most abundant TG species were all identified as being responsible for the discrimination of the hepatic lipid profiles of sedentary and recovering mice in Table 2 and comprised approximately 50% of all detected TGs (Table 4).

**Table 3:** Lipid species with significant alterations in recovering mice found after one-way ANOVA followed by 2-sided Dunnett post hoc.

m/z	t_R_ (min)	Lipid species	Ion adduct	sedentary	run	3 h post (recovering)	run vs. sed	3 h post vs. sed	One-way ANOVA (*p* value)	
				(mean ± SD, µmol/g)	(mean ± SD, µmol/g)	(mean ± SD, µmol/g)	change (%)	change (%)	run vs. sed	3 h postvs. sed
788.619	11.4	PC (36:1)	H+	0.208±0.022	0.206±0.044	0.160±0.021	99	77	0.985	0.009**
812.618	10.9	PC (38:3)	H^+^	0.195±0.020	0.193±0.044	0.150±0.037	99	77	0.987	0.033*
838.634	11.4	PC (40:4)	H^+^	0.017±0.004	0.018±0.007	0.009±0.008	108	52	0.898	0.042*
612.559	12.6	DG (34:1)	NH4+	0.011±0.004	0.010±0.002	0.006±0.006	89	49	0.818	0.036*
846.759	17.8	TG (50:3)	NH4+	0.159±0.046	0.229±0.081	0.296±0.103	143	186	0.173	0.005**
898.789	18.1	TG (54:5)	NH4+	0.270±0.047	0.341±0.076	0.387±0.110	126	143	0.170	0.017*
896.774	17.6	TG (54:6)	NH4+	0.139±0.021	0.161±0.023	0.195±0.046	115	140	0.333	0.004**
901.730	17.6	TG (54:6)	Na^+^	0.021±0.002	0.024±0.003	0.028±0.006	116	136	0.214	0.003**
899.715	16.9	TG (54:7)	Na+	0.015±0.003	0.017±0.004	0.023±0.008	116	155	0.598	0.013*
928.836	19.6	TG (56:4)	NH4+	0.088±0.032	0.098±0.029	0.116±0.036	111	131	0.596	0.031*
952.834	19.1	TG (58:6)	NH4+	0.008±0.010	0.001±0.003	0.022±0.014	15	288	0.330	0.015*
944.773	17.0	TG (58:10)	NH4+	0.017±0.006	0.021±0.004	0.026±0.009	120	150	0.475	0.028*

*p<0.05;

**p<0.01 vs. sedentary mice.

**Table 4:** Summary of most abundant lipids from PC and TG lipid classes.

Lipid species	Sedentary	0 h (run)	3 h post	run vs. sed	3 h post vs. sed	One-way ANOVA (p value)	
	(mean ± SD, µmol/g)	(mean ± SD, µmol/g)	(mean ± SD, µmol/g)	change (%)	change (%)	run vs. sed	3 h post vs. sed
PC (34:1)	0.564±0.244	0.649±0.185	0.587±0.246	115	104	0.6784	0.9695
PC (34:2)	0.568±0.131	0.783±0.314	0.634±0.263	138	112	0.1692	0.8171
PC (36:1)	0.208±0.022	0.206±0.044	0.160±0.021	99	77	0.9851	0.0090**
PC (36:2)	0.501±0.148	0.617±0.148	0.504±0.142	123	101	0.2136	0.9988
PC (36:3)	0.475±0.079	0.529±0.127	0.422±0.128	111	89	0.5448	0.5588
PC (38:4)	0.345±0.143	0.416±0.141	0.371±0.121	120	107	0.4877	0.9017
PC (38:6)	0.365±0.081	0.418±0.079	0.360±0.094	115	99	0.3689	0.9897
PC (40:6)	0.262±0.048	0.274±0.054	0.250±0.057	104	95	0.8765	0.8502
TG (50:1)	0.305±0.093	0.319±0.079	0.343±0.111	105	113	0.9373	0.6396
TG (50:2)	0.362±0.092	0.396±0.091	0.416±0.132	110	115	0.7410	0.4960
TG (52:2)	0.526±0.093	0.491±0.137	0.582±0.182	93	111	0.8444	0.6520
TG (52:3)	0.566±0.083	0.620±0.085	0.698±0.215	109	123	0.6733	0.1356
TG (54:2)	0.222±0.077	0.211±0.055	0.248±0.090	95	112	0.9389	0.7063
TG (54:3)	0.300±0.075	0.32±0.056	0.371±0.103	107	124	0.8365	0.1590
TG (54:4)	0.352±0.077	0.395±0.078	0.447±0.119	112	127	0.5622	0.0945
TG (54:5)	0.270±0.047	0.341±0.076	0.387±0.110	126	143	0.1700	0.0173*

*p<0.05,

**p<0.01 vs. sedentary mice.

The increase in total hepatic TG in the recovery phase was verified in liver tissue lysates using a clinical routine enzymatic method (Fig. 2C). In contrast, in the exercising quadriceps muscle, TG content was decreased and remained lower in the recovery phase (p = 0.05 vs. sedentary mice; Fig. 2D).

### Accumulation of hepatic TG species is correlated with theoretical energy content

The detected hepatic TG species showed differences in their response to acute exercise in the recovery phase that could be related to their content of double bonds (Table 5). TG species containing only saturated acyl chains (TG 46∶0; TG 48∶0; and TG 52∶0) did not accumulate (mean relative change of 90%±40 vs. sedentary mice), and no significant increase was found for TG species containing only one monounsaturated acyl chain (TG 46∶1, TG 48∶1, TG 50∶1, TG 52∶1, TG 54∶1; mean relative change 108%±40 vs. sedentary mice) or two double bonds (TG 46∶2, TG 50∶2, TG 52∶2, TG 54∶2, TG 56∶2; mean relative change 112%±33 vs. sedentary mice). TG species containing more than two and less than seven double bonds showed a significant accumulation in the recovery phase (Table 5). The increase in TG species containing five double bonds was even significant immediately after exercise. Since the data in Table 5 did not consider the contribution of individual TG species to the increase in total TG mass, we calculated the changed relative mass levels of individual TG species by a formula as follows:
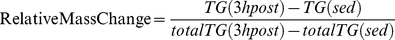



**Table 5:** Summed concentration of detected hepatic TG classes.

Lipid species	Sedentary	run	3 h post (recovering)	run vs. sed	3 h post vs. sed
	(mean ± SD, µmol/g)	(mean ± SD, µmol/g)	(mean ± SD, µmol/g)	change (%)	change (%)
TG 46:0, 48:0, 52:0	0.125±0.024	0.138±0.030	0.113±0.023	111±60	90±40
TG 46:1, 48:1, 50:1, 52:1, 54:1	0.718±0.104	0.725±0.110	0.776±0.119	101±30	108±40
TG 46:2, 50:2, 52:2, 54:2, 56:2	1.446±0.173	1.453±0.169	1.623±0.193	101±22	112±33
TG 48:3, 50:3, 52:3, 54:3, 56:3	1.319±0.184	1.479±0.202	1.700±0.232	112±20	129±33 1)
TG 48:4, 50:4, 52:4, 54:4, 56:4, 58:4	0.845±0.113	0.947±0.126	1.077±0.142	112±25	127±31 1)
TG 50:5, 52:5, 54:5, 56:5	0.557±0.096	0.644±0.122	0.760±0.137	116±20 1)	137±36 1)
TG 52:6, 54:6, 56:6, 58:6	0.316±0.054	0.320±0.061	0.416±0.070	101±21	132±28 2)
TG 54:7, 54:8, 56:7, 56:8, 56:9, 58:7, 58:8, 58:9, 58:10	0.337±0.143	0.359±0.052	0.458±0.200	106±16	136±60

Shown are the summed concentrations of the hepatic TG classes according to Supplemental information, table S1.

1) *p*<0.05 vs. sedentary mice.

2) *p*<0.01 vs. sedentary mice.

**Figure 3:**
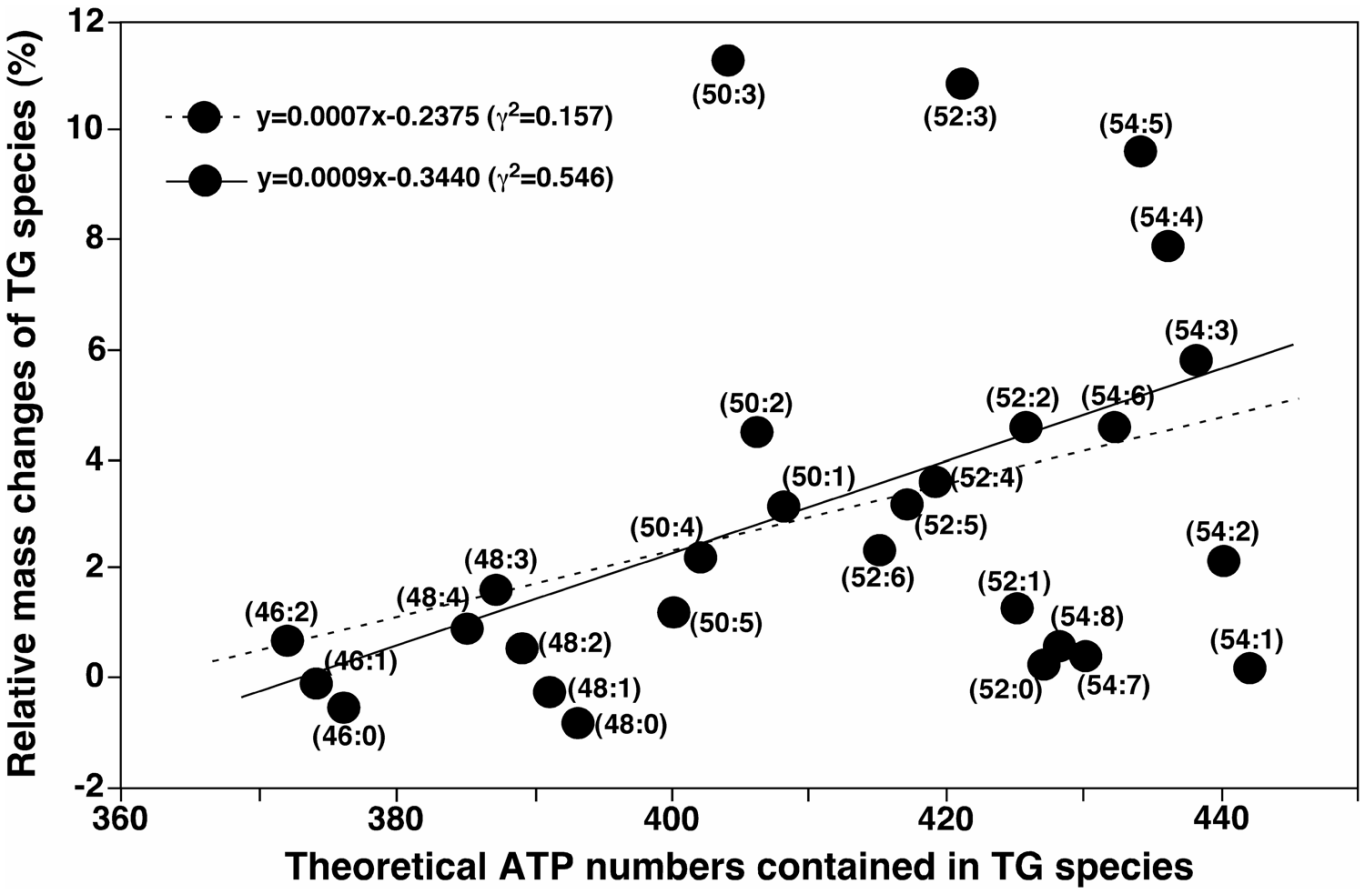
Accumulation of hepatic TG species is correlated with theoretical energy content. Correlation between the changes of the relative mass levels of individual TG species between sedentary and recovering mice and the summed theoretical energies stored in the fatty acyl chains of these TG species. The changes of the relative mass levels of individual TG species were calculated based on the formula shown in the results section. The theoretical energy of each TG species is represented by the numbers of ATP which can be theoretically generated after β-oxidation in mitochondria. The broken line was obtained from the least square linear regression analysis of all data points shown in the figure. The solid line was obtained from the least square linear regression analysis of the data points excluding TG (46:0, 48:0, 52:0, 54:7, 54:8) as discussed in the text.

Moreover, to understand the differences in the accumulation of TG species we analyzed the relationship between the changed relative mass levels of individual TG species and the summed theoretical energies stored in the fatty acyl chains of these TG species. The theoretical energy of each TG species is represented by the numbers of ATP which can be theoretically generated after β-oxidation in mitochondria. We found that the degree of accumulation of individual TG species correlated well with the amount of energy stored in it (Fig. 3). The correlation coefficient (γ^2^) using the least square linear regression analysis is 0.155 for all determined species containing up to 54 carbons and is 0.546 if excluding a few TG species which showed large deviations. These are the TG species containing three saturated fatty acyl chains and those which contain polyunsaturated fatty acyl chain(s) (e.g., TG 54∶7 and TG 54∶8). We should point out that TG species comprising more than 54 carbons were not included in the correlative analysis. This is due to the fact that at least one fatty acyl chain containing 20 or more carbons is needed to build up these species and fatty acids containing 20 or more carbons are always oxidized to 16 or 18 carbons by peroxisomes prior to being oxidized in mitochondria. Therefore, these TG species are no candidates for efficient energy storage.

### Refeeding of fasted mice decreases their hepatic lipid content

**Figure 4:**
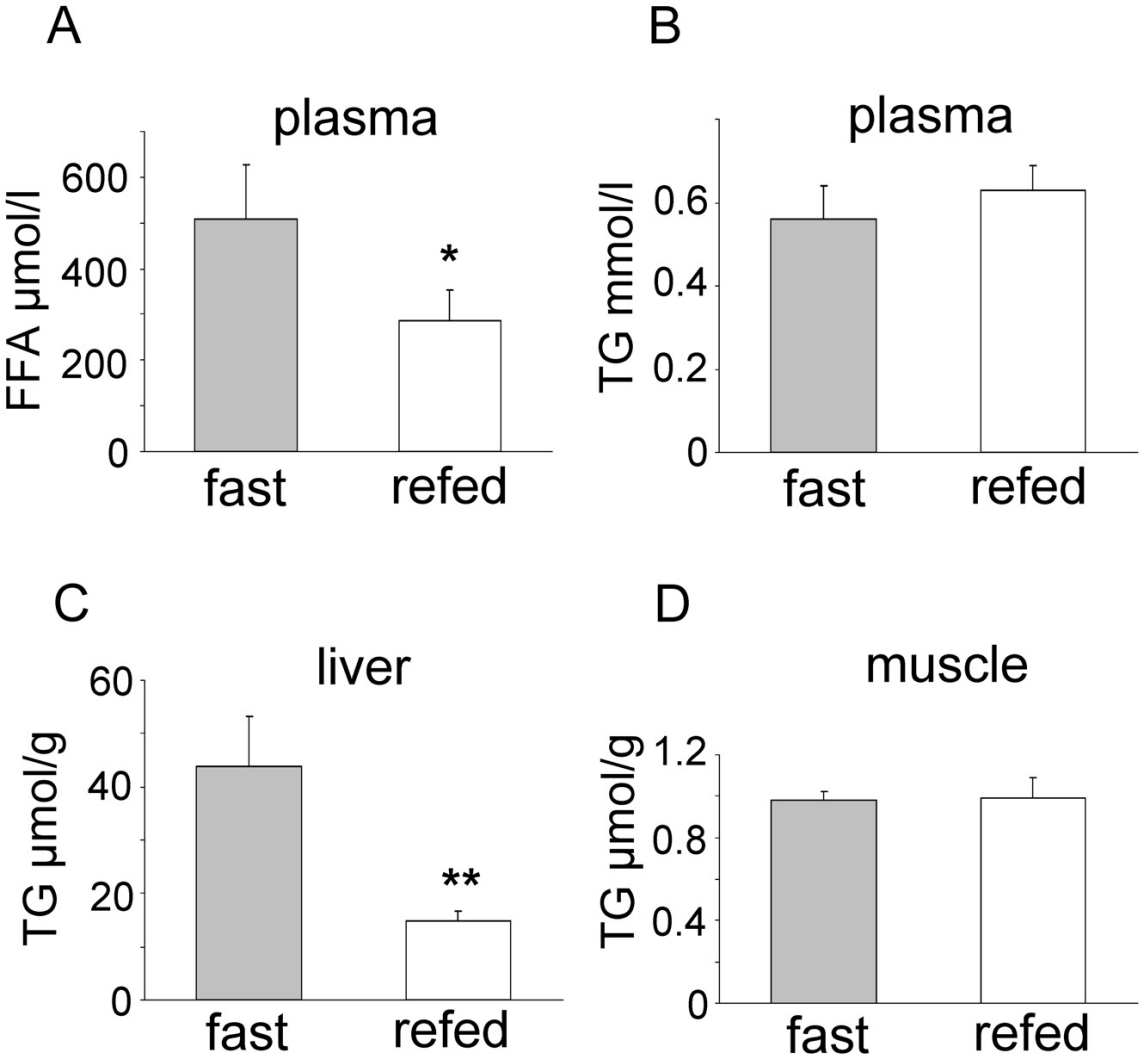
Refeeding of fasted mice decreases their hepatic lipid content. Plasma concentrations of FFA (A) and TG (B) and tissue concentration of TG in liver (C) and gastrocnemius muscle (D) in fasted and refed mice (n = 10 (plasma, liver), n = 6 (muscle), mean ± SEM; * p<0.05; ** p<0.01 vs. fast).

The effects of endurance exercise share some similarities with fasting, particularly on liver metabolism [34]. Acute exercise increases circulating FFA levels, activates hepatic glucose production, and decreases the expression of genes involved in fatty acid synthesis. Since the mice had free access to food in the first hours of the recovery phase, the increased hepatic TG levels might be due to a refeeding phenomenon. We tested this hypothesis by analyzing liver tissue lysates of overnight fasted mice and after 5 h of refeeding. Refed mice had lower circulating FFA levels than fasted mice and similar plasma TG concentrations (Fig. 4A, B). Hepatic TG content of refed mice was similar to that of sedentary mice (14.9±1.7 vs. 16.2±1.7 µmol/g, respectively), while fasted mice had even higher hepatic TG levels than mice after 3 h of recovery period (43.7±9.5 vs. 26.4±2.9 µmol/g, respectively) (Fig. 2C, 4C). TG concentrations measured in gastrocnemius muscles were not different in fasted and refed mice (Fig. 4D). These data indicate a rapid, tissue-specific regulation of the hepatic TG content with no correlation of TG accumulation in the liver and food intake.

## Discussion

A single bout of endurance exercise followed by 3 h recovery led to a significant accumulation of TG in the livers of mice, while the TG content decreased in skeletal muscle. This could be demonstrated by measurement of total TG in liver and muscle tissue using a clinical routine laboratory method as well as by a hepatic lipidomics approach. The analysis of hepatic lipidomics based on UFLC-MS revealed that a large number of lipid species are relevant for the differentiation of the hepatic lipid profile of sedentary and recovering mice. Among the detected LPC, PC, PE, SM, DG, and TG lipid species, TG clearly dominated the pattern of exercise-influenced hepatic lipids under the applied analytical conditions with 17 TG species identified as discriminating lipids in the total lipid pattern analyzed by multivariate data evaluation. Seven of all identified TG species (Table 3) showed significant increases in the recovery phase after one-way ANOVA followed by post hoc with Dunnett.

Although we did not directly address the mechanism leading to the hepatic TG accumulation after exercise, several hints point to an important role of the high availability of plasma FFA during exercise. Prolonged physical activity leads to increased lipolysis of body fat stores and elevated levels of plasma FFA. This high concentration of fatty acids in the circulation might lead not only to increased uptake and oxidation in the working muscle, but also to increased influx into the liver, which might be supported by increased hepatic blood flow in the early recovery phase [35]. Here, fatty acids are reesterified into TG and secreted into circulation in the form of VLDL or stored in the hepatocytes. The accumulation of TG in the liver as a result of high plasma FFA concentrations has been observed during fasting in rodents when the delivery of fatty acids from peripheral fat stores exceeds the oxidative requirements [36], [37]. Similarly we found high hepatic TG levels in overnight fasted mice, which returned to levels of our sedentary control mice after 5 h of refeeding. During prolonged endurance exercise a similar phenomenon might exist, with the secreted FFAs exceeding the oxidative capacity and demands of the working muscle. Therefore it could be speculated that the liver might serve as a buffer reservoir for high FFA plasma levels, which are temporarily stored in the liver as TG and released into circulation when the storage of TG in the adipose tissue is possible and not blocked by hormonal counterregulation. Following that consideration, increases in hepatic TG content should also be detected after long duration of endurance exercise, not only in the early recovery phase. This is confirmed by a previous study that reported on rats performing prolonged exercise and could show a gradual accumulation of TG in the liver depending on the duration of the exercise bout with a doubling at the time of exhaustion [38]. Our exercise protocol of 60 min of treadmill run might be too short to observe such an effect, but also in our study the concentration of almost all TG species was higher immediately after exercise, even though this did not reach statistical significance except the summed increase of TG species containing five double bonds. Therefore we conclude that acute exercise is a stimulus for the accumulation of TG species in the liver similarly to fasting.

A remarkable difference was found in the regulation of hepatic and muscular TG stores. Those of the working muscle appeared to be utilized during running, since the TG concentrations were reduced immediately after exercise, and 3 h of recovery were not sufficient to restore muscle TG concentrations. On the other hand, an overnight fast and 5 h of refeeding did not alter muscle TG content. These data indicate that reliance on endogenous lipid stores induced by caloric restriction or endurance exercise has different effects on the fat depots in liver and muscle and suggest that the hepatic TG pool is under a quite flexible and dynamic control.

The lipidomic analysis clearly demonstrated that the degree of accumulation was not similar for all detected TG but depended on the different molecular species. TG species containing three or two saturated fatty acyl chains did not accumulate. The most reasonable explanation for that are the lipotoxic effects of saturated fatty acids. They have to be detoxified by desaturation [39], [40] or stored as inert TG, which is only possible in an efficient manner together with unsaturated fatty acids [41]. Both detoxifying mechanisms prevent the accumulation of TG containing three saturated fatty acyl chains. Moreover the results suggested that mice have the ability to selectively store TG molecular species that contain high theoretical energy content in the liver after exercise, since a positive correlation was found between TG species with a high relative mass change and their respective theoretical energy content. TG species that must contain at least one polyunsaturated fatty acyl chain (e.g. TG 54∶7) deviate from that correlation. This could be due to the fact that the energy stored in the polyunsaturated fatty acyl chains cannot be efficiently harvested through mitochondrial β-oxidation; instead, they are predominantly oxidized through peroxisomal β-oxidation resulting in the production of thermal energy instead of ATP [42]. A previous report describes a similar selective accumulation of TG species in the liver after a 24 h-fast in rats [43]. TG species containing at least one linoleyl moiety (C 18∶2) showed a net increase in the liver, while TG species containing only saturated and monounsaturated fatty acyl chains decrease. These authors hypothesize that the selective changes in the hepatic TG composition result from a preferential oxidation of saturated and monounsaturated fatty acids in the fasted state [43].

It is also possible that the fatty acid composition of the diet fed to the mice plays a role. The chow has a high content of oleate and linoleate (6.2 and 18.0 g/kg chow, respectively; see Table S2 for details) and this could enhance the storage of TG enriched in these acylmoieties. Our data did not support the hypothesis that food intake per se increases hepatic TG levels, but we could not distinguish whether the source of the accumulated hepatic TG were the dietary lipids or endogenous fatty acids derived from lipolysis. Further experiments using isotope labeled fatty acids would be needed to clarify this point.

The effects of the 60 min treadmill run on other lipid molecular species were less prominent. In general, we found a decrease of some LPC and PC species in the recovery phase. Phospholipids have been discussed as energy source, and short periods of fasting have been shown to lead to substantial decreases in PC and PE species in the heart, but not in muscle [44]. It needs to be evaluated whether some phospholipid species were used as alternate energy source instead of TG in the liver in the recovery phase after endurance exercise. Since exercise acutely reduces VLDL-ApoB secretion in humans [26] and hepatic VLDL secretion has been shown to depend on PC synthesis [45], it would be tempting to speculate that the detected decrease in PC contributes to exercise-induced alterations in lipid metabolism. However, the decreased PC species only contribute to a small percentage of the total hepatic PC pool, therefore an effect on VLDL secretion appears rather speculative. To conclude, the lipidomics analysis did not reveal a pronounced rearrangement in the fatty acid composition of hepatic phospholipids. In contrast, exercise training has been shown to alter the phospholipid profile in rodent or human muscle [46], [47], and to a minor extent of hepatic phospholipids [9].

The current LC-MS lipidomics technology holds great promises in the discovery of potential lipid biomarkers in relation to, for example, hepatic steatosis, disease prevention and health promotion. Its feasibility has been demonstrated in revealing the changes of lipid metabolism in mouse liver after one single bout of exercise. The most prominent effect of acute exercise on hepatic lipids shown here is the induction of hepatic TG storage. Whether this presumably transient hepatic steatosis after exercise is important for the regulatory mechanisms leading to the reduction of hepatic TG levels observed after prolonged training periods remains to be elucidated. But it is possible that exercise might be a physiological challenge for hepatic lipid metabolism similar to fasting that is important for the maintenance of the metabolic flexibility of the liver. A large number of lipid species were responsible for the discrimination of the hepatic lipid pattern of sedentary and recovering mice. These lipids provide a starting point for further understanding of metabolic pathways and investigating integrative biochemical networks.

## Materials and Methods

### Ethics statement

All animal experiments were conducted in accordance with the national guidelines of laboratory animal care and were approved by the local governmental commission for animal research (M2/05, Regierungspraesidium Tübingen, Baden-Württemberg, Germany).

### Chemicals

Liquid chromatography grade chloroform and methanol were purchased from Merck (Darmstadt, Germany). High pressure liquid chromatography grade isopropanol and acetonitrile were from Tedia (Fairfield, OH, USA). Ammonium formate of analytical grade was obtained from Sigma-Aldrich (St.Louis, MO, USA), 0.9% NaCl was from B.Braun (Melsungen, Germany). Triton X-100 was from Sigma (Munich, Germany). Synthetic lipid standards were purchased from Avanti Polar Lipids, Inc. (Alabaster, Alabama, USA) and Sigma-Aldrich (Munich, Germany).

### Animals and exercise

Male C57Bl/6J mice were purchased from The Jackson Laboratory (Bar Harbor, ME, USA) and kept under an inverted light-dark cycle (dark period 9:30–21:30, light period 21:30–9:30) with free access to standard chow (Ssniff, Soest, Germany) and tap water. Fatty acid content of the diet is available as Table S2 in Supporting Information. Exercise experiments were performed between 10:00 and 14:00. Mice were habituated to treadmill running (Mouse Accupacer treadmill with motorized grade adjust, Hugo Sachs Elektronik, March-Hugstetten, Germany) for 10 min at 5 m/min and 5° inclination twice, one and two weeks prior to the experiment.

In the experiments, 12-week-old mice ran 60 min at 14 m/min and 14° uphill slope after 5 min warm-up (5 m/min and 5° inclination) and were either killed immediately after the run or placed back in their cages for 3 h recovery. Sedentary mice remained in their cages. Mice attempting to rest were encouraged to continue running by gently tapping on their back. Mice of all groups had no access to food 60 min before they were killed, either because of running or because of food withdrawal. In fasting/refeeding experiments mice were either fasted overnight or fasted followed by 5 h of free access to food. All animals were anesthetized with an intraperitoneal injection of ketamine (150 mg/kg body weight) and xylazine (10 mg/kg body weight) and killed by decapitation. Livers and muscle tissues were immediately removed and frozen in liquid nitrogen for later processing.

### Plasma biochemical analyses

Glucose was quantified in capillary blood samples taken from the tail vein using an Accu-Chek Aviva glucometer (Roche, Mannheim, Germany). Insulin levels were quantified by radio-immunoassay (Linco Research, St. Charles, MO, USA). FFA and TG were measured in the EDTA-plasma collected after decapitation by fully automatic enzymatic methods on the ADVIA 1650 multi analyzer (Siemens Health Care Diagnostics, Fernwald, Germany). Of note based on the principle of the enzymatic clinical routine assay applied not only TG, but also diacylglyceride (DG), monoacylglyceride and free glycerol are captured.

### Total tissue TG analyses

Frozen liver (100–150 mg) was weighed and homogenized in 1.5 ml of 0.9% NaCl containing 1% Triton X-100 using a TissueLyser (Qiagen, Hilden, Germany). Lysates were clarified by centrifugation at 13,000 g for 10 min and total liver TG content was measured on the ADVIA 1650 multi analyzer. Frozen quadriceps muscle tissue (approximately 100 mg) was weighed, mixed with 0.6 ml cold methanol, 0.13 ml cold water and 50 µl internal standard mixture, and homogenized in a TissueLyser with stainless steel-beads (Qiagen, Hilden, Germany). After adding 0.6 ml chloroform and 0.3 ml water, the lysates were vortexed for 1 min and incubated 10 min on ice. The samples were then centrifuged for 10 min at 13,000 g, 4°C for phase separation. A defined volume of the lower nonpolar layer containing lipids was collected and dried in a vacuum centrifuge. The lipid residue was resuspended in 0.2 ml of 0.9% NaCl containing 1% Triton X-100 and total TG content was measured on the ADVIA 1650 multi analyzer. Determination of hepatic TG content gave similar results with each method.

### Hepatic lipidomics analysis

#### Preparation of lipid standards

Stock solutions of lipid standards were separately dissolved in organic solvent. Briefly, LPC (17∶0), PE (34∶0) and PC (34∶0) were separately dissolved in a chloroform/methanol mixture (2∶1, v/v) and TG (51∶0) was dissolved in chloroform by weighing an exact amount of each lipid standard in new glass vials. The stock solutions were stored at −20°C until further use. To prepare the working solution, a certain volume of each stock solution was allowed to reach room temperature, transferred into a new glass vial resulting in a mixture 1∶1∶1∶1 (v/v/v/v) and vortexed. The working solution was then diluted 1∶10 in a chloroform/methanol mixture (2∶1, v/v) for same-day use to the final concentration used for internal standard addition. See Table S3 in Supporting Information for details of the four internal lipid standards.

#### Liver lipid extraction

Frozen liver pieces (approximately 100 mg) of eight individual mice per group were homogenized and the lipids were extracted as described above for quadriceps muscle tissue. The lipid residue was resuspended in 0.3 ml of chloroform/methanol (2∶1, v/v) followed by 20 times dilution with acetonitrile/isopropanol/water (65∶30∶5, v/v/v) for lipidomics analysis.

#### Ultra fast liquid chromatography coupled to ion-trap time-of-flight mass spectrometry (UFLC−IT-TOF−MS)

The diluted lipid extracts were analyzed on a hybrid ion-trap time-of-flight mass spectrometer (Shimadzu, Kyoto, Japan) coupled with an ultra fast liquid chromatography system (Shimadzu, Kyoto, Japan). The column was an Ascentis® Express C_8_ 2.1×150 mm packed with fused-core 2.7 µm diameter particles (Sigma-Aldrich, Munich, Germany). Each sample was analyzed twice. The UFLC separation of hepatic lipids was achieved according to the previously published method [32]. The liver lipid profiling was carried out on Shimadzu IT-TOF-MS equipped with an electrospray ion source in the positive ion mode. The voltages of the interface and the detector of the TOF analyzer were 4.5 kV and 1.6 kV, respectively. The temperatures of the curved desorption line and heat block were both set at 200°C. The flow rate of the nebulizing gas was 1.5 L/min. The dry gas pressure was 0.2 MPa. The flight tube temperature was stable at 40°C and the ion trap pressure was maintained 1.6×10^−2^ Pa. Ultra-high purity argon was used for collision and ion cooling. The data were collected at mass range of m/z 400−1500 with an ion accumulation of 20 ms using LCMS solution software (Shimadzu, Kyoto, Japan).

#### Data processing

LC−MS raw data were pre-processed by the Phenomenome Profiler M Series version 2.5 (AM+, Canada). Briefly, raw data were first converted to CDF format and then subjected to peak picking. The parameters for peak picking were smoothing points, 3; peak noise calculation, cutoff of 0.1% base peak. The picked peaks were then aligned using Profiler's clustering algorithm based on the retention time and m/z of the peaks. After that, a matrix for multivariate data analysis was generated by the software with a mass clustering window of 0.005 Da and the retention time clustering window of 0.2 min. The matrix covered the information of the retention time, m/z and the ion intensity for each picked peak. Specifically, if certain ion intensity was less than peak noise value in one sample, zero would be assigned to that ion intensity in the matrix. The obtained data set was processed by the “80% rule”[48], that is, only the ions with intensities above zero present in at least 80% of either group were included for further data analyses. Subsequently internal standard correction according to the strategy described previously [32], normalization to mouse liver weight, and averaging the duplicate injections was performed. Partial least squares discriminant analysis (PLS-DA) was subsequently applied as a supervised modeling method to the pareto-scaled data to visualize possible relations between the samples (scores plot) and possible relations between lipids (loading plot) related to the samples and the study groups using SIMCA-P 11.0 (Umetrics AB, Umeå, Sweden). The discriminating individual lipid molecular species of the PLS-DA model were selected by the S-plot [49].

### Statistical analysis

Data are expressed as means ± SEM. Unless indicated otherwise one-way ANOVA followed by the Dunnett post hoc test was applied to assess the statistical significance between groups using SPSS statistics (version 17, SPSS Inc., USA). Statistical significance was set at p<0.05.

## Supporting Information

Table S1 Detailed information of the detected 115 lipid species.
(0.35 MB DOC)

Table S2 Relative fatty acid content of the standard chow.
(0.03 MB DOC)

Table S3 Detailed information of four lipid internal standards.
(0.03 MB DOC)
