# Supplementary material for: Lipidomics Analysis Reveals Efficient Storage of Hepatic Triacylglycerides Enriched in Unsaturated Fatty Acids after One Bout of Exercise in Mice
Source: PLoS One. 2010 Oct 13;5(10):e13318. doi: 10.1371/journal.pone.0013318 (PMC2954156; doi:10.1371/journal.pone.0013318)
Supplement: Table S1 — Detailed information of the detected 115 lipid species. (0.35 MB DOC) [file pone.0013318.s001.doc]

**Table S1 Detailed information of the detected 115 lipid species**

| No. | Lipid species | tR (min) | adduct ion | observed | theoretical | error | sedentary | 0h (run) | 3h post | run vs. sed | 3 h post vs. sed | One-way ANOVA (p value) | |
| --- | --- | --- | --- | --- | --- | --- | --- | --- | --- | --- | --- | --- | --- |
|  |  |  |  | m/z | m/z | (ppm) | (mean ± SD, µmol/g) | (mean ± SD, µmol/g) | (mean ± SD, µmol/g) | change (%) | change (%) | run vs. sed | 3h post vs. sed |
| 1 | LPC (16:0) | 2.48 | H+ | 496.3406 | 496.3398 | 1.61 | 0.153 ± 0.023 | 0.154 ± 0.042 | 0.137 ± 0.032 | 100 | 90 | 0.9990 | 0.5327 |
| 2 | LPC (18:0) | 3.22 | H+ | 524.3720 | 524.3711 | 1.72 | 0.123 ± 0.032 | 0.126 ± 0.047 | 0.109 ± 0.043 | 102 | 89 | 0.9867 | 0.7308 |
| 3 | LPC (18:1) | 2.63 | H+ | 522.3567 | 522.3554 | 2.49 | 0.040 ± 0.007 | 0.034 ± 0.025 | 0.027 ± 0.013 | 85 | 68 | 0.7066 | 0.2527 |
| 4 | LPC (18:2) | 2.19 | H+ | 520.3417 | 520.3398 | 3.65 | 0.039 ± 0.006 | 0.032 ± 0.023 | 0.030 ± 0.013 | 81 | 75 | 0.5481 | 0.3781 |
| 5 | LPC (20:4) | 2.12 | H+ | 544.3417 | 544.3398 | 3.49 | 0.026 ± 0.011 | 0.016 ± 0.018 | 0.019 ± 0.013 | 61 | 73 | 0.2900 | 0.5151 |
| 6 | LPC (O-18:2) | 3.22 | H+ | 506.3632 | 506.3605 | 5.33 | 0.045 ± 0.019 | 0.047 ± 0.02 | 0.043 ± 0.019 | 105 | 94 | 0.9663 | 0.9503 |
| 7 | PC (32:0) | 10.18 | H+ | 734.5729 | 734.5694 | 4.76 | 0.186 ± 0.028 | 0.206 ± 0.041 | 0.173 ± 0.035 | 111 | 93 | 0.4363 | 0.6686 |
| 8 | PC (32:1) | 9.41 | H+ | 732.5596 | 732.5538 | 7.92 | 0.098 ± 0.028 | 0.109 ± 0.049 | 0.074 ± 0.033 | 111 | 75 | 0.8049 | 0.3481 |
| 9 | PC (32:2) | 8.70 | H+ | 730.5420 | 730.5381 | 5.34 | 0.047 ± 0.013 | 0.052 ± 0.014 | 0.043 ± 0.015 | 110 | 90 | 0.7177 | 0.7305 |
| 10 | PC (34:1) | 10.36 | H+ | 760.5885 | 760.5851 | 4.47 | 0.564 ± 0.244 | 0.649 ± 0.185 | 0.587 ± 0.246 | 115 | 104 | 0.6784 | 0.9695 |
| 11 | PC (34:2) | 9.60 | H+ | 758.5728 | 758.5694 | 4.48 | 0.568 ± 0.131 | 0.783 ± 0.314 | 0.634 ± 0.263 | 138 | 112 | 0.1692 | 0.8171 |
| 12 | PC (34:3) | 9.01 | H+ | 756.5578 | 756.5538 | 5.29 | 0.159 ± 0.025 | 0.165 ± 0.045 | 0.150 ± 0.031 | 103 | 94 | 0.9312 | 0.8193 |
| 13 | PC (34:4) | 8.45 | H+ | 754.5430 | 754.5381 | 6.49 | 0.015 ± 0.003 | 0.014 ± 0.003 | 0.013 ± 0.005 | 97 | 90 | 0.9727 | 0.6976 |
| 14 | PC (36:1) | 11.38 | H+ | 788.6187 | 788.6164 | 2.92 | 0.208 ± 0.022 | 0.206 ± 0.044 | 0.160 ± 0.021 | 99 | 77 | 0.9851 | 0.0090 |
| 15 | PC (36:2) | 10.59 | H+ | 786.6032 | 786.6007 | 3.18 | 0.501 ± 0.148 | 0.617 ± 0.148 | 0.504 ± 0.142 | 123 | 101 | 0.2136 | 0.9988 |
| 16 | PC (36:3) | 9.86 | H+ | 784.5867 | 784.5851 | 2.04 | 0.475 ± 0.079 | 0.529 ± 0.127 | 0.422 ± 0.128 | 111 | 89 | 0.5448 | 0.5588 |
| 17 | PC (36:4) | 9.66 | H+ | 782.5672 | 782.5694 | -2.81 | 0.119 ± 0.166 | 0.226 ± 0.142 | 0.181 ± 0.167 | 190 | 153 | 0.3192 | 0.6507 |
| 18 | PC (36:5) | 8.43 | H+ | 780.5567 | 780.5538 | 3.72 | 0.053 ± 0.025 | 0.074 ± 0.047 | 0.089 ± 0.026 | 142 | 170 | 0.3587 | 0.0800 |
| 19 | PC (36:6) | 8.32 | H+ | 778.5410 | 778.5381 | 3.72 | 0.006 ± 0.004 | 0.008 ± 0.005 | 0.005 ± 0.005 | 139 | 92 | 0.5815 | 0.9764 |
| 20 | PC (38:2) | 11.62 | H+ | 814.6349 | 814.6320 | 3.56 | 0.046 ± 0.017 | 0.055 ± 0.022 | 0.040 ± 0.012 | 120 | 88 | 0.4914 | 0.7540 |
| 21 | PC (38:3) | 10.86 | H+ | 812.6178 | 812.6164 | 1.72 | 0.195 ± 0.020 | 0.193 ± 0.044 | 0.149 ± 0.037 | 99 | 77 | 0.9873 | 0.0327 |
| 22 | PC (38:4) | 10.46 | H+ | 810.6004 | 810.6007 | -0.37 | 0.345 ± 0.143 | 0.416 ± 0.141 | 0.371 ± 0.121 | 120 | 107 | 0.4877 | 0.9017 |
| 23 | PC (38:5) | 9.74 | H+ | 808.5819 | 808.5851 | -3.96 | 0.050 ± 0.044 | 0.048 ± 0.013 | 0.032 ± 0.025 | 97 | 64 | 0.9925 | 0.4050 |
| 24 | PC (38:6) | 9.07 | H+ | 806.5701 | 806.5694 | 0.87 | 0.365 ± 0.081 | 0.418 ± 0.079 | 0.360 ± 0.094 | 115 | 99 | 0.3689 | 0.9897 |
| 25 | PC (38:7) | 8.38 | H+ | 804.5548 | 804.5538 | 1.24 | 0.065 ± 0.010 | 0.067 ± 0.026 | 0.055 ± 0.015 | 103 | 85 | 0.9685 | 0.4814 |
| 26 | PC (40:4) | 11.42 | H+ | 838.6337 | 838.6320 | 2.03 | 0.017 ± 0.004 | 0.018 ± 0.007 | 0.009 ± 0.008 | 108 | 52 | 0.8984 | 0.0421 |
| 27 | PC (40:5) | 10.84 | H+ | 836.6177 | 836.6164 | 1.55 | 0.010 ± 0.002 | 0.012 ± 0.002 | 0.009 ± 0.002 | 116 | 94 | 0.1866 | 0.7535 |
| 28 | PC (40:6) | 10.12 | H+ | 834.6019 | 834.6007 | 1.44 | 0.262 ± 0.048 | 0.274 ± 0.054 | 0.250 ± 0.057 | 104 | 95 | 0.8765 | 0.8502 |
| 29 | PC (40:7) | 9.36 | H+ | 832.5840 | 832.5851 | -1.32 | 0.143 ± 0.024 | 0.152 ± 0.033 | 0.140 ± 0.034 | 107 | 98 | 0.7658 | 0.9774 |
| 30 | PC (40:8) | 8.68 | H+ | 830.5717 | 830.5694 | 2.77 | 0.048 ± 0.004 | 0.055 ± 0.017 | 0.051 ± 0.016 | 115 | 106 | 0.4670 | 0.8827 |
| 31 | PE (34:2) | 9.76 | H+ | 716.5297 | 716.5225 | 10.05 | 0.050 ± 0.016 | 0.057 ± 0.023 | 0.049 ± 0.024 | 113 | 98 | 0.7777 | 0.9952 |
| 32 | PE (36:1) | 11.43 | H+ | 746.5743 | 746.5694 | 6.56 | 0.007 ± 0.004 | 0.006 ± 0.005 | 0.006 ± 0.004 | 97 | 83 | 0.9914 | 0.8139 |
| 33 | PE (36:2) | 10.72 | H+ | 744.5597 | 744.5538 | 7.92 | 0.053 ± 0.011 | 0.059 ± 0.022 | 0.051 ± 0.018 | 112 | 96 | 0.7096 | 0.9563 |
| 34 | PE (36:3) | 9.92 | H+ | 742.5443 | 742.5381 | 8.35 | 0.033 ± 0.008 | 0.039 ± 0.010 | 0.038 ± 0.010 | 120 | 116 | 0.2984 | 0.4206 |
| 35 | PE (36:4) | 9.66 | H+ | 740.5297 | 740.5225 | 9.72 | 0.032 ± 0.031 | 0.039 ± 0.046 | 0.022 ± 0.021 | 121 | 68 | 0.8972 | 0.7734 |
| 36 | PE (36:5) | 8.93 | H+ | 738.5120 | 738.5068 | 7.04 | 0.014 ± 0.002 | 0.016 ± 0.006 | 0.011 ± 0.003 | 114 | 77 | 0.5707 | 0.2455 |
| 37 | PE (38:3) | 10.94 | H+ | 770.5742 | 770.5694 | 6.23 | 0.019 ± 0.004 | 0.020 ± 0.006 | 0.017 ± 0.003 | 104 | 92 | 0.9221 | 0.7527 |
| 38 | PE (38:4) | 10.61 | H+ | 768.5580 | 768.5538 | 5.46 | 0.159 ± 0.064 | 0.191 ± 0.105 | 0.148 ± 0.057 | 120 | 93 | 0.6440 | 0.9374 |
| 39 | PE (38:5) | 9.89 | H+ | 766.5428 | 766.5381 | 6.13 | 0.058 ± 0.021 | 0.063 ± 0.036 | 0.054 ± 0.019 | 109 | 92 | 0.8898 | 0.9216 |
| 40 | PE (38:6) | 9.26 | H+ | 764.5289 | 764.5225 | 8.37 | 0.111 ± 0.037 | 0.131 ± 0.058 | 0.119 ± 0.047 | 118 | 107 | 0.6273 | 0.9316 |
| 41 | PE (38:7) | 8.54 | H+ | 762.5117 | 762.5068 | 6.43 | 0.014 ± 0.003 | 0.016 ± 0.004 | 0.012 ± 0.002 | 113 | 86 | 0.3737 | 0.2905 |
| 42 | PE (40:4) | 11.59 | H+ | 796.5876 | 796.5851 | 3.14 | 0.008 ± 0.001 | 0.009 ± 0.005 | 0.004 ± 0.004 | 109 | 51 | 0.9048 | 0.0944 |
| 43 | PE (40:6) | 10.28 | H+ | 792.5530 | 792.5538 | -1.01 | 0.065 ± 0.039 | 0.078 ± 0.023 | 0.079 ± 0.026 | 120 | 121 | 0.6102 | 0.5635 |
| 44 | PE (40:7) | 9.54 | H+ | 790.5389 | 790.5381 | 1.01 | 0.011 ± 0.012 | 0.015 ± 0.015 | 0.017 ± 0.016 | 142 | 161 | 0.7569 | 0.5643 |
| 45 | SM (d18:0/24:0) | 13.23 | H+ | 815.7029 | 815.7001 | 3.43 | 0.072 ± 0.008 | 0.071 ± 0.033 | 0.057 ± 0.01 | 98 | 79 | 0.9891 | 0.2610 |
| 46 | SM (d18:0/24:1) | 12.26 | H+ | 813.6857 | 813.6844 | 1.6 | 0.100 ± 0.014 | 0.125 ± 0.031 | 0.105 ± 0.018 | 125 | 105 | 0.0677 | 0.8574 |
| 47 | SM (d18:0/24:2) | 11.42 | H+ | 811.6606 | 811.6688 | -10.1 | 0.011 ± 0.002 | 0.015 ± 0.006 | 0.011 ± 0.003 | 133 | 101 | 0.1233 | 0.9958 |
| 48 | SM (d18:1/22:0) | 12.22 | H+ | 787.6715 | 787.6688 | 3.43 | 0.111 ± 0.03 | 0.104 ± 0.035 | 0.087 ± 0.018 | 93 | 79 | 0.8283 | 0.1978 |
| 49 | SM (d18:1/23:0) | 12.76 | H+ | 801.6873 | 801.6844 | 3.62 | 0.02 ± 0.003 | 0.020 ± 0.012 | 0.016 ± 0.005 | 102 | 79 | 0.9937 | 0.4456 |
| 50 | SM (d18:1/16:0) | 9.12 | H+ | 703.5798 | 703.5749 | 6.96 | 0.028 ± 0.006 | 0.025 ± 0.015 | 0.024 ± 0.007 | 89 | 85 | 0.7856 | 0.6101 |
| 51 | SM (d18:1/20:0) | 11.19 | H+ | 759.6352 | 759.6375 | -3.03 | 0.009 ± 0.001 | 0.007 ± 0.006 | 0.006 ± 0.004 | 71 | 69 | 0.3300 | 0.2886 |
| 52 | DG (34:1) | 12.60 | NH4+ | 612.5585 | 612.5562 | 3.75 | 0.011 ± 0.004 | 0.010 ± 0.002 | 0.006 ± 0.006 | 89 | 49 | 0.8185 | 0.0359 |
| 53 | DG (34:2) | 11.84 | NH4+ | 610.5429 | 610.5405 | 3.93 | 0.040 ± 0.01 | 0.045 ± 0.009 | 0.04 ± 0.014 | 113 | 101 | 0.5739 | 0.9982 |
| 54 | DG (36:2) | 12.74 | NH4+ | 638.5744 | 638.5718 | 4.07 | 0.014 ± 0.003 | 0.013 ± 0.008 | 0.013 ± 0.004 | 97 | 95 | 0.9800 | 0.9479 |
| 55 | DG (36:3) | 12.03 | NH4+ | 636.5588 | 636.5562 | 4.08 | 0.041 ± 0.009 | 0.042 ± 0.006 | 0.04 ± 0.009 | 104 | 97 | 0.9158 | 0.9239 |
| 56 | DG (36:4) | 11.25 | NH4+ | 634.5420 | 634.5405 | 2.36 | 0.024 ± 0.005 | 0.027 ± 0.006 | 0.028 ± 0.007 | 115 | 120 | 0.4330 | 0.2196 |
| 57 | TG (46:0) | 18.24 | NH4+ | 796.7431 | 796.7389 | 5.27 | 0.019 ± 0.009 | 0.022 ± 0.016 | 0.013 ± 0.01 | 113 | 69 | 0.8769 | 0.5153 |
| 58 | TG (46:1) | 17.56 | NH4+ | 794.7269 | 794.7232 | 4.66 | 0.029 ± 0.018 | 0.032 ± 0.017 | 0.028 ± 0.015 | 111 | 96 | 0.8922 | 0.9856 |
| 59 | TG (46:2) | 16.86 | NH4+ | 792.7112 | 792.7076 | 4.54 | 0.024 ± 0.026 | 0.035 ± 0.016 | 0.032 ± 0.032 | 148 | 136 | 0.5830 | 0.7353 |
| 60 | TG (48:0) | 19.07 | NH4+ | 824.7743 | 824.7702 | 4.97 | 0.067 ± 0.032 | 0.079 ± 0.048 | 0.058 ± 0.025 | 118 | 86 | 0.7351 | 0.8290 |
| 61 | TG (48:1) | 18.38 | NH4+ | 822.7588 | 822.7545 | 5.23 | 0.106 ± 0.052 | 0.116 ± 0.049 | 0.103 ± 0.048 | 110 | 97 | 0.8735 | 0.9914 |
| 62 | TG (48:2) | 17.66 | NH4+ | 820.7427 | 820.7389 | 4.63 | 0.112 ± 0.056 | 0.124 ± 0.046 | 0.119 ± 0.062 | 111 | 106 | 0.8610 | 0.9525 |
| 63 | TG (48:2) | 17.66 | Na+ | 825.6991 | 825.6943 | 5.81 | 0.020 ± 0.010 | 0.025 ± 0.011 | 0.020 ± 0.010 | 125 | 100 | 0.5261 | 1.0000 |
| 64 | TG (48:3) | 16.97 | NH4+ | 818.7263 | 818.7232 | 3.79 | 0.068 ± 0.043 | 0.084 ± 0.025 | 0.089 ± 0.060 | 123 | 130 | 0.7121 | 0.5698 |
| 65 | TG (48:4) | 16.40 | NH4+ | 816.7108 | 816.7076 | 3.92 | 0.009 ± 0.015 | 0.016 ± 0.009 | 0.020 ± 0.022 | 185 | 236 | 0.5717 | 0.2727 |
| 66 | TG (50:1) | 19.22 | NH4+ | 850.7885 | 850.7858 | 3.17 | 0.305 ± 0.093 | 0.319 ± 0.079 | 0.343 ± 0.111 | 105 | 113 | 0.9373 | 0.6396 |
| 67 | TG (50:1) | 19.22 | Na+ | 855.7455 | 855.7412 | 5.02 | 0.044 ± 0.009 | 0.048 ± 0.011 | 0.049 ± 0.013 | 108 | 112 | 0.7555 | 0.5451 |
| 68 | TG (50:2) | 18.50 | NH4+ | 848.7734 | 848.7702 | 3.77 | 0.362 ± 0.092 | 0.396 ± 0.091 | 0.416 ± 0.132 | 110 | 115 | 0.7410 | 0.4960 |
| 69 | TG (50:2) | 18.50 | Na+ | 853.7299 | 853.7256 | 5.04 | 0.046 ± 0.004 | 0.050 ± 0.009 | 0.051 ± 0.014 | 109 | 111 | 0.6019 | 0.5233 |
| 70 | TG (50:3) | 17.80 | NH4+ | 846.7589 | 846.7545 | 5.2 | 0.159 ± 0.046 | 0.229 ± 0.081 | 0.296 ± 0.103 | 143 | 186 | 0.1727 | 0.0050 |
| 71 | TG (50:3) | 17.80 | Na+ | 851.7138 | 851.7099 | 4.58 | 0.045 ± 0.006 | 0.048 ± 0.010 | 0.048 ± 0.012 | 106 | 106 | 0.8046 | 0.8164 |
| 72 | TG (50:4) | 17.08 | NH4+ | 844.7422 | 844.7389 | 3.91 | 0.128 ± 0.047 | 0.160 ± 0.050 | 0.156 ± 0.042 | 124 | 121 | 0.3219 | 0.4088 |
| 73 | TG (50:5) | 16.49 | NH4+ | 842.7271 | 842.7232 | 4.63 | 0.025 ± 0.016 | 0.033 ± 0.007 | 0.040 ± 0.032 | 130 | 159 | 0.6977 | 0.2899 |
| 74 | TG (52:0) | 20.76 | NH4+ | 880.8236 | 880.8328 | -10.44 | 0.038 ± 0.017 | 0.037 ± 0.013 | 0.042 ± 0.019 | 97 | 109 | 0.9818 | 0.8881 |
| 75 | TG (52:1) | 20.06 | NH4+ | 878.8206 | 878.8171 | 3.98 | 0.172 ± 0.065 | 0.165 ± 0.050 | 0.188 ± 0.076 | 96 | 109 | 0.9658 | 0.8413 |
| 76 | TG (52:2) | 19.34 | NH4+ | 876.8052 | 876.8015 | 4.22 | 0.526 ± 0.093 | 0.491 ± 0.137 | 0.582 ± 0.182 | 93 | 111 | 0.8444 | 0.6520 |
| 77 | TG (52:2) | 19.34 | Na+ | 881.7613 | 881.7569 | 4.99 | 0.055 ± 0.006 | 0.052 ± 0.012 | 0.059 ± 0.031 | 95 | 107 | 0.9433 | 0.8839 |
| 78 | TG (52:3) | 18.70 | NH4+ | 874.7894 | 874.7858 | 4.12 | 0.566 ± 0.083 | 0.620 ± 0.085 | 0.698 ± 0.215 | 109 | 123 | 0.6733 | 0.1356 |
| 79 | TG (52:3) | 18.70 | Na+ | 879.7464 | 879.7412 | 5.91 | 0.061 ± 0.01 | 0.056 ± 0.014 | 0.049 ± 0.048 | 92 | 81 | 0.9300 | 0.6492 |
| 80 | TG (52:4) | 18.17 | NH4+ | 872.7754 | 872.7702 | 5.96 | 0.174 ± 0.033 | 0.180 ± 0.034 | 0.218 ± 0.057 | 104 | 125 | 0.9356 | 0.0917 |
| 81 | TG (52:4) | 18.17 | Na+ | 877.7313 | 877.7256 | 6.49 | 0.027 ± 0.007 | 0.022 ± 0.005 | 0.037 ± 0.021 | 81 | 136 | 0.6549 | 0.2608 |
| 82 | TG (52:5) | 17.46 | NH4+ | 870.7596 | 870.7545 | 5.86 | 0.129 ± 0.033 | 0.139 ± 0.02 | 0.168 ± 0.046 | 108 | 130 | 0.7753 | 0.0655 |
| 83 | TG (52:5) | 17.46 | Na+ | 875.7161 | 875.7099 | 7.08 | 0.022 ± 0.005 | 0.026 ± 0.006 | 0.030 ± 0.012 | 118 | 141 | 0.5461 | 0.0855 |
| 84 | TG (52:6) | 16.76 | NH4+ | 868.7439 | 868.7390 | 5.67 | 0.057 ± 0.024 | 0.066 ± 0.011 | 0.086 ± 0.040 | 116 | 150 | 0.7443 | 0.0937 |
| 85 | TG (54:1) | 20.90 | NH4+ | 906.8529 | 906.8484 | 4.96 | 0.062 ± 0.036 | 0.045 ± 0.016 | 0.065 ± 0.033 | 73 | 104 | 0.4237 | 0.9810 |
| 86 | TG (54:2) | 20.19 | NH4+ | 904.8367 | 904.8328 | 4.31 | 0.222 ± 0.077 | 0.211 ± 0.055 | 0.248 ± 0.090 | 95 | 112 | 0.9389 | 0.7063 |
| 87 | TG (54:2) | 20.19 | Na+ | 909.7930 | 909.7882 | 5.28 | 0.029 ± 0.010 | 0.028 ± 0.007 | 0.034 ± 0.013 | 98 | 117 | 0.9933 | 0.5363 |
| 88 | TG (54:3) | 19.51 | NH4+ | 902.8201 | 902.8171 | 3.32 | 0.300 ± 0.075 | 0.32 ± 0.056 | 0.371 ± 0.103 | 107 | 124 | 0.8365 | 0.1590 |
| 89 | TG (54:3) | 19.52 | Na+ | 907.7775 | 907.7725 | 5.51 | 0.037 ± 0.007 | 0.040 ± 0.010 | 0.043 ± 0.012 | 107 | 117 | 0.8183 | 0.3543 |
| 90 | TG (54:4) | 18.80 | NH4+ | 900.8051 | 900.8015 | 4 | 0.352 ± 0.077 | 0.395 ± 0.078 | 0.447 ± 0.119 | 112 | 127 | 0.5622 | 0.0945 |
| 91 | TG (54:4) | 18.80 | Na+ | 905.7617 | 905.7569 | 5.3 | 0.044 ± 0.005 | 0.047 ± 0.010 | 0.045 ± 0.018 | 107 | 103 | 0.8114 | 0.9630 |
| 92 | TG (54:5) | 18.08 | NH4+ | 898.7891 | 898.7858 | 3.67 | 0.270 ± 0.047 | 0.341 ± 0.076 | 0.387 ± 0.110 | 126 | 143 | 0.1700 | 0.0173 |
| 93 | TG (54:5) | 18.08 | Na+ | 903.7452 | 903.7412 | 4.43 | 0.035 ± 0.003 | 0.041 ± 0.010 | 0.046 ± 0.015 | 118 | 133 | 0.4031 | 0.0717 |
| 94 | TG (54:6) | 17.60 | NH4+ | 896.7742 | 896.7702 | 4.46 | 0.139 ± 0.021 | 0.161 ± 0.023 | 0.195 ± 0.046 | 115 | 140 | 0.3329 | 0.0043 |
| 95 | TG (54:6) | 17.60 | Na+ | 901.7302 | 901.7256 | 5.1 | 0.021 ± 0.002 | 0.024 ± 0.003 | 0.028 ± 0.006 | 116 | 136 | 0.2144 | 0.0029 |
| 96 | TG (54:7) | 16.90 | NH4+ | 894.7546 | 894.7545 | 0.11 | 0.034 ± 0.020 | 0.035 ± 0.008 | 0.039 ± 0.012 | 102 | 114 | 0.9902 | 0.7178 |
| 97 | TG (54:7) | 16.90 | Na+ | 899.7153 | 899.7099 | 6 | 0.015 ± 0.003 | 0.017 ± 0.004 | 0.023 ± 0.008 | 116 | 155 | 0.5975 | 0.0128 |
| 98 | TG (54:8) | 16.53 | NH4+ | 892.7412 | 892.7389 | 2.58 | 0.020 ± 0.011 | 0.022 ± 0.005 | 0.028 ± 0.017 | 107 | 136 | 0.9628 | 0.3725 |
| 99 | TG (56:2) | 21.00 | NH4+ | 932.8678 | 932.8641 | 3.97 | 0.051 ± 0.029 | 0.039 ± 0.014 | 0.062 ± 0.036 | 77 | 121 | 0.6210 | 0.6691 |
| 100 | TG (56:3) | 20.32 | NH4+ | 930.8514 | 930.8484 | 3.22 | 0.082 ± 0.040 | 0.083 ± 0.028 | 0.106 ± 0.062 | 102 | 130 | 0.9973 | 0.4746 |
| 101 | TG (56:4) | 19.61 | NH4+ | 928.8363 | 928.8328 | 3.77 | 0.088 ± 0.032 | 0.098 ± 0.029 | 0.115 ± 0.036 | 111 | 131 | 0.7699 | 0.1947 |
| 102 | TG (56:4) | 19.62 | Na+ | 933.7902 | 933.7882 | 2.14 | 0.012 ± 0.003 | 0.013 ± 0.004 | 0.016 ± 0.004 | 113 | 138 | 0.5957 | 0.0307 |
| 103 | TG (56:5) | 19.19 | NH4+ | 926.8194 | 926.8171 | 2.48 | 0.076 ± 0.017 | 0.064 ± 0.030 | 0.088 ± 0.043 | 84 | 116 | 0.6759 | 0.6500 |
| 104 | TG (56:6) | 18.20 | NH4+ | 924.8033 | 924.8015 | 1.95 | 0.091 ± 0.019 | 0.068 ± 0.045 | 0.084 ± 0.057 | 75 | 93 | 0.4850 | 0.9313 |
| 105 | TG (56:7) | 17.26 | NH4+ | 922.7776 | 922.7858 | -8.89 | 0.030 ± 0.016 | 0.023 ± 0.003 | 0.040 ± 0.024 | 77 | 135 | 0.6358 | 0.3723 |
| 106 | TG (56:8) | 17.23 | NH4+ | 920.7708 | 920.7702 | 0.65 | 0.064 ± 0.057 | 0.076 ± 0.047 | 0.090 ± 0.059 | 118 | 140 | 0.8837 | 0.5454 |
| 107 | TG (56:8) | 17.23 | Na+ | 925.7298 | 925.7256 | 4.54 | 0.013 ± 0.002 | 0.012 ± 0.002 | 0.010 ± 0.007 | 94 | 80 | 0.9218 | 0.3821 |
| 108 | TG (56:9) | 16.69 | NH4+ | 918.7575 | 918.7545 | 3.27 | 0.026 ± 0.010 | 0.029 ± 0.005 | 0.038 ± 0.019 | 113 | 150 | 0.8276 | 0.0953 |
| 109 | TG (58:4) | 20.40 | NH4+ | 956.8570 | 956.8641 | -7.42 | 0.014 ± 0.011 | 0.016 ± 0.009 | 0.022 ± 0.012 | 113 | 159 | 0.9221 | 0.2401 |
| 110 | TG (58:6) | 19.10 | NH4+ | 952.8344 | 952.8328 | 1.68 | 0.008 ± 0.010 | 0.001 ± 0.003 | 0.022 ± 0.013 | 15 | 288 | 0.3299 | 0.0148 |
| 111 | TG (58:7) | 18.28 | NH4+ | 950.8131 | 950.8171 | -4.21 | 0.003 ± 0.005 | 0.005 ± 0.005 | 0.010 ± 0.007 | 166 | 340 | 0.7405 | 0.0503 |
| 112 | TG (58:8) | 18.20 | NH4+ | 948.8012 | 948.8015 | -0.32 | 0.033 ± 0.017 | 0.030 ± 0.006 | 0.035 ± 0.022 | 92 | 106 | 0.9210 | 0.9641 |
| 113 | TG (58:9) | 17.50 | NH4+ | 946.7881 | 946.7858 | 2.43 | 0.066 ± 0.025 | 0.077 ± 0.009 | 0.086 ± 0.038 | 118 | 131 | 0.5997 | 0.2386 |
| 114 | TG (58:9) | 17.50 | Na+ | 951.7462 | 951.7412 | 5.25 | 0.008 ± 0.004 | 0.011 ± 0.002 | 0.010 ± 0.005 | 126 | 121 | 0.4146 | 0.5436 |
| 115 | TG (58:10) | 17.00 | NH4+ | 944.7726 | 944.7702 | 2.54 | 0.017 ± 0.006 | 0.021 ± 0.004 | 0.026 ± 0.009 | 120 | 150 | 0.4753 | 0.0282 |
