# Supplementary material for: Lipidomics Analysis Reveals Efficient Storage of Hepatic Triacylglycerides Enriched in Unsaturated Fatty Acids after One Bout of Exercise in Mice
Source: PLoS One. 2010 Oct 13;5(10):e13318. doi: 10.1371/journal.pone.0013318 (PMC2954156; doi:10.1371/journal.pone.0013318)
Supplement: Table S2 — Relative fatty acid content of the standard chow. (0.03 MB DOC) [file pone.0013318.s002.doc]

**Table S2. Relative fatty acid content of the standard chow**

| **Fatty acid species** | **g/kg chow** |
| --- | --- |
| C 14:0 | 0.1 |
| C 16:0 | 4.7 |
| C 16:1 | 0.1 |
| C 18:0 | 0.8 |
| C18:1 | 6.2 |
| C 18:2 | 18.0 |
| C18:3 | 2.3 |
| C 20:0 | 0.1 |
| C 20:1 | 0.2 |
