# Supplementary material for: Lipidomics Analysis Reveals Efficient Storage of Hepatic Triacylglycerides Enriched in Unsaturated Fatty Acids after One Bout of Exercise in Mice
Source: PLoS One. 2010 Oct 13;5(10):e13318. doi: 10.1371/journal.pone.0013318 (PMC2954156; doi:10.1371/journal.pone.0013318)
Supplement: Table S3 — Detailed information of four lipid internal standards. (0.03 MB DOC) [file pone.0013318.s003.doc]

**Table S3 D**etailed information of four lipid internal standards

| **Lipid internal standard** | **protonated ion**  **[M + H]^+^** | **ammoniated ion**  **[M + NH_4_]^+^** | **concentration** | | | |
| --- | --- | --- | --- | --- | --- | --- |
|  |  |  | **stock (mg/ml)** | **working (µg/ml)** | **final (µg/ml)** | **spiking level (µg /sample)** |
| LPC (17:0) | 510.3557 |  | 2.4 | 600 | 60 | 3 |
| PE (34:0) | 720.5583 |  | 4.4 | 1100 | 110 | 5.5 |
| PC (34:0) | 762.6025 |  | 7.2 | 1800 | 180 | 9 |
| TG (51:0) |  | 866.8209 | 8.8 | 2200 | 220 | 11 |
